# Prevalence and factors associated with probable depression among the oldest old during the Covid‐19 pandemic: evidence from the large, nationally representative ‘Old Age in Germany (D80+)’ study

**DOI:** 10.1111/psyg.13129

**Published:** 2024-05-03

**Authors:** André Hajek, Hans‐Helmut König, Angelina R. Sutin, Antonio Terracciano, Martina Luchetti, Yannick Stephan, Razak M. Gyasi

**Affiliations:** ^1^ Department of Health Economics and Health Services Research University Medical Center Hamburg‐Eppendorf, Hamburg Center for Health Economics Hamburg Germany; ^2^ College of Medicine Florida State University Tallahassee Florida USA; ^3^ Euromov, University of Montpellier Montpellier France; ^4^ African Population and Health Research Center Nairobi Kenya; ^5^ National Centre for Naturopathic Medicine, Faculty of Health Southern Cross University Lismore New South Wales Australia

**Keywords:** aged, 80 and above, depression, institutionalization, mental health, migration, morbidity, oldest old, spousal loss, self‐rated health

## Abstract

**Background:**

To date, most studies examining the prevalence and determinants of depression among individuals aged 80 and over have used geographically limited samples that are not generalisable to the wider population. Thus, our aim was to identify the prevalence and the factors associated with probable depression among the oldest old in Germany based on nationally representative data.

**Methods:**

Data were taken from the nationally representative ‘Old Age in Germany (D80+)’ study (*n* = 8386; November 2020 to April 2021) covering both community‐dwelling and institutionalised individuals aged 80 and over. The Short Form of the Depression in Old Age Scale was used to quantify probable depression.

**Results:**

Probable depression was found in 40.7% (95% CI: 39.5% to 42.0%) of the sample; 31.3% were men (95% CI: 29.7% to 32.9%) and 46.6% women (95% CI: 44.9% to 48.3%). The odds of probable depression were positively associated with being female (odds ratio (OR): 1.55, 95% CI: 1.30 to 1.84), being divorced (compared to being married, OR: 1.33, 95% CI: 1.01 to 1.76), being widowed (OR: 1.14, 95% CI: 1.00 to 1.30), having a low education (e.g., medium education compared to low education, OR: 0.86, 95% CI: 0.74 to 0.99), living in an institutionalised setting (OR: 2.36, 95% CI: 1.84 to 3.02), living in East Germany (OR: 1.21, 95% CI, 1.05 to 1.39), not having German citizenship (German citizenship compared to other citizenship, OR: 0.55, 95% CI: 0.31 to 0.95), poor self‐rated health (OR: 0.31, 95% CI: 0.28 to 0.34), and the number of chronic conditions (OR: 1.12, 95% CI: 1.09 to 1.14).

**Conclusion:**

About four out of 10 individuals aged 80 and over in Germany had probable depression, underlining the importance of this challenge. Knowledge of specific risk factors for this age group may assist in addressing older adults at risk of probable depression.

## INTRODUCTION

Germany's demographic landscape is expected to change markedly over the next few decades. The population of the oldest old (i.e., individuals aged 80 years and over) is expected to increase significantly, driven by, among other things, improvements in health care and a rising life expectancy.^1^ This demographic shift comes with a range of challenges, with individuals in this age bracket facing many hurdles: some might lose their mobility, have a higher risk of falling, experience decreases in sensory abilities, and lose their friends and acquaintances.^2^ Such obstacles can contribute to an increased risk of depression.^3^, ^4^, ^5^ And indeed, depression has been found to increase with ageing.^6^ Depression has deleterious implications for a range of outcomes, including frailty,^7^ dementia^8^ and suicidality.^9^ Furthermore, depression can increase healthcare costs.^10^

A recently published systematic review and meta‐analysis revealed a global overall point prevalence of depression of 35.1% (95% CI: 30.2% to 40.4%) among adults 65 years and older.^11^ A previous study showed a prevalence of 20.0% among the general adult population (18 to 70 years) in Germany in August/September 2021^12^ based on the Patient Health Questionnaire‐9.^13^ However, the prevalence rate varied according to age group (from 31.8% among individuals aged 18 to 29 years to 11.8% among individuals aged 60 to 70 years).^12^ The prevalence of probable depression among the oldest old in Germany remains unclear.

Moreover, some recent studies have investigated sociodemographic and health‐related factors associated with depression among the oldest old. For instance, one study emphasised the role of spousal loss for depression among the oldest old in six large German cities.^14^ Other research also demonstrated the role of geographical location in risk for developing depression among individuals aged 85 years and over.^15^ A further study showed that age was positively associated with depression among individuals aged 80 years and over residing in the Hainan Province in the south of China.^16^ Higher depression rates were also observed among women aged 90 years compared to men in this age bracket, using data from Tuscany (Mugello area, location in Italy).^17^ Another recent study also stressed the role of functional decline in developing depressive symptoms in North‐Rhine Westphalia (the most populous state in Germany).^18^ However, to date, most studies examining the determinants of depression focused on individuals aged 80 and over have used geographically limited samples that are not generalisable to this age group in the respective countries.

Therefore, due to limited knowledge in this area, the objective of this study was to identify the prevalence of probable depression in Germany and to determine the factors associated with it, based on a nationally representative sample of individuals aged 80 years and over. Exploring the prevalence and the factors associated with probable depression among the oldest old is of great importance. A better understanding of the mental health needs of the oldest old is crucial for developing targeted interventions and supportive healthcare systems.

## METHODS

### Sample

Data were taken from the Old Age in Germany (D80+) study. This study investigates a large, nationwide representative sample of individuals aged 80 years and over living in Germany. Both community‐dwelling and institutionalised individuals were included, which is a key feature of the D80+ study. The University of Cologne, in collaboration with Cologne Centre for Ethics, Rights, Economics, and Social Sciences of Health (ceres) and the German Centre of Gerontology (DZA), carried out the research. It was funded by the Federal Ministry for Family Affairs, Senior Citizens, Women and Youth (BMFSFJ).

The study design required to be modified because of the pandemic. Face‐to‐face interviews were intended, but data collection was required to be carried out through written postal surveys (self‐completion, PAPI; subsequent and telephone interviews (CATI) taking place from mid‐May to mid‐October 2021). The written interview included questions that were considered to be of the highest priority within the study, whereas the telephone interviews included lower priority content (telephone interviews: also high priority content, which can only be collected with the aid of interviews such as cognitive testing). The determination of high and lower priority content was carried out by ceres. They anticipated that the response rate for the written interview would surpass that of the in‐depth telephone survey. Hence, they preferred to capture as much crucial content as possible within the written interview. At the same time, it was acknowledged that, the questionnaire's length should not exceed the previous iteration's length, so as to enhance participation willingness. Consequently, central themes and constructs (e.g., sociodemographic factors, health, housing or family situation), especially those pertinent to the life circumstances of individuals aged 80 years and over, and the research's main focuses, were primarily addressed in the written interview. Accordingly, the survey content of the telephone interview holds a predominantly secondary significance (e.g., personality or critical life events).

The fieldwork, data collection, and data weighting were carried out by the Institut für angewandte Sozialwissenschaft GmbH (Infas), located in Bonn (Germany). Details on methodology are in the corresponding method report.^19^ A short description of the D80+ (and other details) is also available in English language (URL: https://www.dza.de/en/research/fdz/d80/documentation).

In total, more than 10 000 individuals participated in the written interview. Due to missing values (please see Appendix S1 for further details), the analytical sample equalled *n* = 8386 individuals. Data collection for the written interview took place from late November 2020 to late April 2021. Further details are provided by Albrecht *et al*.^20^ Probable depression was only assessed in the written postal survey.

### Dependent variable: depression

Depressive symptoms were assessed with the Short Form of the Depression in Old Age Scale (DIA‐S4).^21^, ^22^ It consists of four items (each: no (0) or yes (1)). For each question, the time frame assessed was the last 14 days. An exemplary item is: ‘Do you feel depressed?’ One item (‘Can you enjoy your life, even if some things are harder for you?’) was recoded. A sum score was calculated based on the four items. Three items focus on main symptoms of depression (loss of pleasure, low energy and depressed mood), in line with the International Classification of Diseases (ICD‐10).^23^ One item refers to rumination. A negatively worded item was used to avoid acquiescence bias.^24^

The cumulative score of this tool ranges from zero to four. Higher values reflect more depressive symptoms. The favourable psychometric characteristics of the DIA‐S4 have been demonstrated elsewhere.^21^, ^22^ Similar to prior research,^24^ Cronbach's alpha was 0.66 in our current study (McDonald's omega was 0.67). Prior research^24^ suggested a cut‐off score of 1.5 for indicating depression (sensitivity: 87%; specificity: 68%). This cut‐off score was used to differentiate between the presence and absence of probable depression.

### Independent variables

Guided by former research (e.g., Vink et al.^25^) and theoretical deliberations, we selected the independent variables for regression analysis. The following sociodemographic factors were selected for logistic regression analysis: sex (men; women), age group (80 to 84 years; 85 to 89 years; 90 years and over), marital status (married; married, but living separated from spouse; divorced; widowed; single), living situation (private household; institutionalised setting), education (low education; medium education; high education; based on the International Standard Classification of education 2011^26^), region (East Germany; West Germany), and citizenship (German citizenship; other citizenship).

The following health‐related factors were also included in regression analysis: self‐rated health, and a count score for the number of chronic conditions (in each case: absence of the respective disease: zero; one otherwise). A single item was used to quantify self‐rated health. The exact wording was: ‘How would you generally describe your state of health in the last 4 weeks?’ (very bad; rather bad; rather good; very good).

The following 21 chronic conditions were included in the count score: myocardial infarction; heart failure; hypertension; stroke; mental illness; cancer; diabetes, respiratory or lung disease; back pain; gastrointestinal disease; kidney disease; liver disease; blood disease (e.g., anaemia); joint or bone disease; bladder disease; sleep disorders; eye disease or visual impairment; ear disease or hearing impairment; neurological disease; (blood) vascular disease; thyroid disease. This tool is based on the multimorbidity index in old age.^27^, ^28^ It may be worth noting that the chronic condition *mental illness* was only weakly correlated with probable depression (Cramer's *V* = 0.25). Therefore, we also included it in the count score for chronic conditions.

### Statistical analysis

Sample characteristics for the analytical sample were calculated first, followed by the prevalence of probable depression, overall and by sociodemographic groups (sex, age group, family status, living situation, education, region, and citizenship). Multiple logistic regressions were used to investigate the factors associated with probable depression among the oldest old. Cluster‐robust standard errors were computed (based on the primary sampling unit) because of the multistage sampling procedure. Sampling weights were applied to adjust for survey non‐response and the disproportion sampling design (referring to the oversampling of older age groups and men).^19^ The Stata tool ‘omegacoef’ was used to compute McDonald's omega.^29^

Statistical significance was set at *P* < 0.05. Statistical analyses were conducted using Stata 18.0 (Stata Corp., College Station, TX, USA).

## RESULTS

### Sample characteristics and prevalence rates

Sample characteristics for the analytical sample (*n* = 8386) are detailed in Table 1. In total, 61.7% of the participants were female, and most participants (58.9%) were in the 80 to 84 age group (average age: 85.5, SD: 4.1, 80 to 100 years). Furthermore, 22.9% of participants had a low education. More details are presented in Table 1.

**Table 1:** Sample characteristics of the analytical sample with *n* = 8386 individuals (weighted)

Variables	Mean (SD) / *n* (%)
Sex	
Men	3212 (38.3%)
Women	5174 (61.7%)
Age group	
80 to 84 years	4938 (58.9%)
85 to 89 years	2243 (26.7%)
90 years and over	1204 (14.4%)
Marital status	
Married	3392 (40.5%)
Married, living separated from spouse	77 (0.9%)
Divorced	407 (4.9%)
Widowed	4163 (49.6%)
Single	346 (4.1%)
Living situation	
Private household	7505 (89.5%)
Institutionalised setting	881 (10.5%)
Education	
Low education	1920 (22.9%)
Medium education	4341 (51.8%)
High education	2126 (25.3%)
Region	
West Germany	6527 (77.8%)
East Germany	1859 (22.2%)
Citizenship	
Other citizenship	57 (0.7%)
German citizenship	8329 (99.3%)
Number of chronic conditions (based on 21 chronic conditions)	4.6 (2.7)
Self‐rated health (from 1 = very poor to 4 = very good)	2.6 (0.7)

The prevalence rates (also by subgroup) are detailed in Table 2. Overall, 40.7% (95% CI: 39.5% to 42.0%) of participants had probable depression (men: 31.3%, 95% CI: 29.7% to 32.9%; women: 46.6%, 95% CI: 44.9% to 48.3%). There were differences in the prevalence of probable depression between the age groups, with higher rates in the older participants (e.g., among individuals aged 90 years and over: 53.6%, 95% CI: 50.8% to 56.4%).

**Table 2:** Prevalence rates (total sample and for several sociodemographic groups)

Group	Presence of probable depression (95% CI)
Total sample	40.7% (39.5% to 42.0%)
Gender	
Male	31.3% (29.7% to 32.9%)
Female	46.6% (44.9% to 48.3%)
Age group	
80–84	36.3% (34.6% to 37.9%)
85–89	43.8% (41.6% to 46.0%)
90+	53.6% (50.8% to 56.4%)
Family status	
Married	33.2% (31.6% to 34.8%)
Married, living separated from spouse	43.5% (31.7% to 56.1%)
Divorced	43.7% (38.1% to 49.4%)
Widowed	46.1% (44.2% to 48.0%)
Single	45.0% (38.0% to 52.2%)
Living situation	
Private household	37.6% (36.4% to 38.8%)
Institutionalised setting	67.6% (62.6% to 72.2%)
Education	
Low education	49.3% (46.7% to 51.9%)
Medium education	41.0% (39.2% to 42.7%)
High education	31.7% (29.5% to 33.9%)
Region	
West Germany	39.7% (38.3% to 41.1%)
East Germany	44.5% (41.8% to 47.2%)
Citizenship	
Other citizenship	43.7% (34.7% to 53.1%)
German citizenship	40.7% (39.4 to 42.0%)

There were particularly high differences in the prevalence rates between participants living in a private household (37.6%, 95% CI: 36.4% to 38.8%) and individuals residing in an institutionalised setting (67.6%, 95% CI: 62.6% to 72.2%). Further details are provided in Table 2.

### Regression analysis

The results of the multiple logistic regression (with probable depression vs. absence of probable depression as outcome) are presented in Table 3. Pseudo *R* ^2^ equaled 0.17. The odds of probable depression were positively associated with being female (odds ratio (OR): 1.55, 95% CI: 1.30 to 1.84), being divorced (compared to being married, OR: 1.33, 95% CI: 1.01 to 1.76), being widowed (OR: 1.14, 95% CI: 1.00 to 1.30), living in an institutionalised setting (OR: 2.36, 95% CI: 1.84 to 3.02), having a low education (e.g., medium education compared to low education, OR: 0.86, 95% CI: 0.74 to 0.99), living in East Germany (OR: 1.21, 95% CI, 1.05 to 1.39), not having German citizenship (German citizenship compared to other citizenship, OR: 0.55, 95% CI: 0.31 to 0.95), poor self‐rated health (OR: 0.31, 95% CI: 0.28 to 0.34) and the number of chronic conditions (OR: 1.12, 95% CI: 1.09 to 1.14). Age group was not significantly associated with the odds of probable depression. More details are in Table 3.

**Table 3:** Determinants of probable depression. Results of multiple logistic regressions

Independent variables	Probable depression
Sex: women (Ref.: men)	1.55***
	(1.30–1.84)
Age group: ‐ 85–89 (Ref.: 80 to 84 years)	0.96
	(0.80–1.14)
‐ 90 and over	1.01
	(0.83–1.23)
Marital status: married, living separated from spouse (Ref.: married)	1.17
	(0.64–2.14)
‐ Divorced	1.33*
	(1.01–1.76)
‐ Widowed	1.14*
	(1.00–1.30)
‐ Single	1.18
	(0.85–1.64)
Living situation: institutionalised setting (Ref.: private household)	2.36***
	(1.84–3.02)
Education: medium education (Ref.: low education)	0.86*
	(0.74–0.99)
‐ High education	0.71***
	(0.59–0.85)
Region: East Germany (Ref.: West Germany)	1.21**
	(1.05–1.39)
Citizenship: German citizenship (Ref.: other citizenship)	0.55*
	(0.31–0.95)
Self‐rated health (from 1 = very poor to 4 = very good)	0.31***
	(0.28–0.34)
Number of chronic conditions (based on 21 chronic conditions)	1.12***
	(1.09–1.14)
Constant	10.92***
	(5.82–20.48)
Observations	8386
Pseudo *R* ^2^	0.17

Odds ratios are displayed; 95% CI in parentheses; cluster‐robust standard errors were computed (based on the primary sampling unit); sampling weights were used; it was also adjusted for sample cells used for the stratification of the secondary sampling unit.

*** *P* < 0.001;

** *P* < 0.01;

* *P* < 0.05;

^†^ *P* < 0.10.

## DISCUSSION

Our aim was to identify the prevalence and the factors associated with probable depression among the oldest old in Germany. Key among the findings was that over four out of 10 individuals aged 80 and over had probable depression in Germany. Women had roughly 50% greater risk of depression compared to men. The prevalence also varied depending on the living situation, with about double the risk of probable depression for adults in institutionalised settings. Poor self‐rated health and the number of chronic conditions were also associated with a greater odds of probable depression. Finally, older adults with a more disadvantaged background, such as non‐citizens, people with lower education or from East Germany, were also more likely to have probable depression.

A former study^12^ identified a prevalence of 20% among adults 18 to 70 years in Germany in late summer 2021 (individuals aged 60 to 70 years: 11.8%). This is in stark contrast to our current study. Several factors may explain these discrepancies: differences in (1) the tools used, (2) the time period in which the study was undertaken and (3) differences in the age group. For example, the former study used the screening tool Patient Health Questionnaire‐9 to quantify probable depression,^13^ whereas our current study used DIA‐S4, which was specifically designed for use in old age. However, comparable prevalence studies using the DIA‐S4 are lacking. The former study was also restricted to individuals up to 70 years, while our study focused specifically on community‐dwelling and institutionalised individuals aged 80 years and over. Additionally, while the previous study^12^ focused on the time period of August/September 2021, the data collection for the written interview in our study took place between November 2020 and April 2021. This period in late 2020 and early 2021 was characterised by a partial lockdown (including several Covid‐19‐related restrictions) in Germany, whereas most restrictions had been eased by late summer 2021 (e.g., due to Covid‐19 vaccinations). Three‐G rules (i.e., public spaces were only open to those who are vaccinated, recovered, or tested) were applied in numerous places in late summer 2021.

Our sample consisted of a very vulnerable group, especially at the height of the pandemic, which had to face many contact restrictions, particularly in nursing homes. These contact restrictions could have had a very negative impact on mental health.^30^, ^31^ A potentially great coronavirus anxiety (due to the potentially severe course of the disease in the event of an infection, particularly before the coronavirus vaccination was introduced in Germany at the end of 2020) could also be of great importance among individuals in this age bracket. These factors could be closely linked to a greater likelihood of probable depression.^32^

It should be acknowledged that the prevalence of probable depression may be overestimated due to the low specificity. However, these results are consistent with previous research in the United States that found an increase in depressive symptoms in older adulthood, particularly after age of 80.^6^

Similar to other studies on the link between sex and probable depression among older adults,^33^, ^34^ we found higher odds among women in this age bracket. This may be partly attributed to biological differences, such as higher levels of serotonergic, inflammatory, and neurotrophic markers.^33^ Moreover, women may be more likely to outlive friends and relatives, which could also play a role. Grief, feelings of loss, and loneliness could therefore be relevant.^35^ Furthermore, differences in physical activity between older women and men^36^ may be of importance here.

While basic descriptive statistics suggested that the older participants had more depressive symptoms, age was not a significant predictor in the regression model (when other factors were constant). This pattern indicates that health‐related factors and common, normative events in older adulthood were more important than age. Specifically, being widowed and divorced were positively associated with probable depression. This extends previous research regarding spousal loss and the development of depression in Germany.^14^ Spousal loss is a very critical life event. It could reflect the loss of a marriage, which is often a big part of a person's life in these birth cohorts in Germany.^37^ This may change life profoundly and result in probable depression.

Another critical life event may be entering a nursing home. Older adults often prefer to live at home for as long as possible.^38^, ^39^ Life in a nursing home (often caused by functional impairment^40^) could be perceived as less fulfilling in many respects (e.g., perceived autonomy or contact with friends and relatives^41^) compared to life in a private home. This could explain the higher odds of probable depression^42^ among individuals living in an institutionalised setting.

Furthermore, having a lower education was associated with higher odds of probable depression in our study. A meta‐analysis examined the association between education and risk for late‐life depression and found that less education was associated with an increased risk of late‐life depression.^43^ This association can be attributed to many factors, including lower levels of general self‐efficacy and cognitive function among individuals with lower education.^43^ It has been shown that self‐efficacy is associated with depression.^44^ Other potential explanatory mechanisms refer to a higher involvement in health‐risk behaviours such as alcohol use or smoking and less involvement in physical activity among less educated individuals.^45^, ^46^ Moreover, lower education is linked to higher inflammation, which may increase the risk of depression.^47^

Individuals not having German citizenship (compared to individuals with German citizenship) had higher odds of probable depression. We assume that individuals not having German citizenship commonly have direct migration experiences. The potentially traumatic experiences before migration and the challenges of living in another country can contribute to poor mental health.^48^ Feelings of exclusion, discrimination, or racism^49^ may also contribute to depression.^50^ Moreover, these individuals could be anxious about the well‐being of friends and family residing in their home countries.^51^, ^52^ They may also feel helpless and sad as they are generally too frail to visit their loved ones from their home countries (or receive visitors), and this would have been particularly true during the pandemic.^51^, ^52^ Furthermore, increased prevalence may be attributed to concerns arising from inadequate understanding of the coronavirus, stemming from language barriers.^12^

Living in East Germany (compared to West Germany) was associated with higher odds of probable depression. There was a tendency for more migration to the west in Germany after reunification, with young, single adults, or highly educated individuals most likely to migrate westward.^53^ Oldest old individuals in the east may miss the geographical proximity to friends and relatives (e.g., their children). Higher loneliness levels in East Germany have also been found by Buecker *et al*.^54^ Buecker *et al*. partly attributed this to the thinner settlement pattern and increased distance to regional centres in certain parts of East Germany.^54^ Such feelings could have a negative impact on mental health.^55^ It may also be partly due to having lived as a young adult in a ‘different’ political system with fewer resources and opportunities.

Regarding health‐related factors, poor self‐rated health and a higher number of chronic conditions were both associated with higher odds of probable depression. Poor health can reflect limitations in activities of daily living.^56^ Such a resulting lack of social activities due to functional impairment^57^ may contribute to higher odds of depression.^58^ Moreover, depression is often a comorbid condition for severe health conditions, such as cancer and neurodegenerative diseases.^59^

Some strengths and caveats of this present work are worth acknowledging. Data were taken from a large, nationally representative sample of individuals aged 80 and over. Furthermore, in the D80+ study, both individuals residing in private households as well as individuals residing in institutionalised settings were included. Additionally, we used an established screening tool to quantify probable depression. Nevertheless, future research based on more sophisticated tools is recommended. It is worth noting that the D80+ study had a cross‐sectional design. In addition, the use of antidepressants was not taken into account in the D80+ study.^60^ Furthermore, factors such as quality of the marital relationship were also not assessed in the D80+ study. Additionally, this study design has limitations regarding directionality and causal inferences, and future work needs to evaluate its generalisability to populations outside Germany.

## CONCLUSIONS AND FUTURE RESEARCH

About four out of 10 individuals aged 80 and over in Germany had probable depression, underlining the significance and importance of this challenge. Knowledge of the factors associated with probable depression may assist in addressing individuals at risk of probable depression. Future longitudinal studies may focus on the stability of depression among the oldest old, and on the social and health consequences of depression. Moreover, the impact of modifiable factors (e.g., lifestyle‐related factors) on the chances of developing depression could be examined, as well as modifiable factors associated with remission.

## AUTHOR CONTRIBUTIONS

AH: conceptualisation, data curation, methodology, project administration, visualisation; roles/writing ‐ original draft, writing ‐ review and editing, formal analysis. HHK: conceptualisation, resources, writing ‐ review and editing, visualisation. ARS: conceptualisation, writing ‐ review and editing, visualisation. AT: conceptualisation, writing ‐ review and editing, visualisation. ML: conceptualisation, writing ‐ review and editing, visualisation. YS: conceptualisation, writing ‐ review and editing, visualisation. RMG: conceptualisation, writing ‐ review and editing, supervision, visualisation. All authors have read and agreed to the published version of the manuscript.

## Supporting information


**Data S1.** Supporting appendix.

## Data Availability

The data that support the findings of this study are available from DZA. Restrictions apply to the availability of these data, which were used under license for this study. Data are available from https://www.dza.de/en/research/fdz/access‐to‐data/application with the permission of DZA.
